# Differential effects of chronotype on physical activity and cognitive performance in older adults

**DOI:** 10.3389/fepid.2023.1029221

**Published:** 2023-04-17

**Authors:** Hilary Hicks, Kayla Meyer, Amber Watts

**Affiliations:** ^1^ Department of Psychology, University of Kansas, Lawrence, KS, United States; ^2^ Alzheimer’s Disease Research Center, University of Kansas Medical Center, Fairway, KS, United States

**Keywords:** chronotype, sleep, physical activity, actigraphy, cognition, older adults

## Abstract

**Introduction:**

Chronotypes reflect individuals' preferred activity and sleep patterns (e.g., “morning-types” vs. “evening-types”) and are associated with health and physical activity. Less is known about the relationship between chronotype and cognitive health in older adults. It is unclear whether chronotype's influence is driven by sleep timing or disruption. This study explored the relationship between chronotype, physical activity, and cognitive performance in older adults with and without self-reported sleep disorders.

**Methods:**

Participants were 153 older adults (*M *= 70.35, *SD *= 5.89) who wore an Actigraph on the non-dominant wrist for seven days to measure total physical activity, peak physical activity, and chronotype (sleep interval midpoint). We categorized participants as morning-, evening-, and intermediate-chronotypes and assessed cognitive performance in domains of attention, executive function, and verbal memory.

**Results:**

MANCOVAs showed patterns of activity across the 24-hour day differed between chronotypes such that morning-types were active earlier and evening-types active later, *p*s > .001. Total physical activity and average peak activity did not differ between chronotypes, (*p*s* *≥ .117). Timing of peak activity followed expectations (morning-types peaked earliest (*p* = .019). Evening-types exhibited significantly worse executive function and attention than intermediate-types, *p *= .008. When excluding participants with sleep disorders, evening-types engaged in significantly less total physical activity than other groups, but cognitive performance did not differ.

**Discussion:**

We found no differences in total or peak physical activity between groups, which is inconsistent with findings from studies in younger samples. This suggests the role of chronotype on physical activity may change with age and points to the potential impact of methodological discrepancies. While evening-types exhibited worse executive function and attention performance, this finding disappeared when participants with sleep disorders were excluded. Sleep dysregulation rather than sleep timing may be driving this difference. Recent trends in physical activity research explore activity patterns across the 24-hour day and acknowledge codependence between different activity types. Our findings suggest chronotype and activity timing may be important as researchers advance this line of research in older adults.

## Introduction

Advancing age is an unavoidable and significant risk factor for cognitive decline and the onset of neurodegenerative disease (1). However, cognitive decline is not an inevitable part of aging. Significant effort has been dedicated to promoting and maintaining cognitive wellness with aging through engagement in health behaviors known to mitigate risk. One health behavior, physical activity, is associated with reduced risk of cognitive decline (2). Evidence suggests a dose-response relationship between one type of physical activity (exercise) and cognitive performance (3), with some amount of activity conferring benefits and greater amounts of activity conferring greater benefits. Despite these benefits, few older adults meet recommended guidelines of physical activity and are instead sedentary for more than 8.5 h of the waking day on average (4). In response, significant research efforts have focused on increasing older adults' engagement in physical activity through the design and implementation of targeted exercise interventions.

The timing of physical activity can differentially impact the benefits of activity for the health of the body. For example, the reduction in blood pressure commonly seen post-exercise may be greater when performed in the morning compared to the evening. Evening exercise, on the other hand, appears better suited to building muscles and burning fat while morning exercise may be better for prevention of muscle loss (5). The increased secretion of cortisol, a common marker of stress in response to exercise, may take longer to return to baseline when exercise is performed in the evening compared to the morning despite identical intensity and duration of activity (6). Of note, research that investigates the relationship between impacts of physical activity and time-of-day does not commonly employ older adult participants, so it is unclear whether these findings extend to an older population.

The fluctuating effects of physical activity on body physiology can be attributed to the circadian rhythm, which is the approximately 24-hour cycle exhibited by certain physiological processes in the human body [e.g., core body temperature, melatonin and cortisol secretion, and sleep (7);]. Light and other zeitgebers (environmental signals that orient us to time) can influence this process such as nutritional intake, social demands, and physical activity. The circadian rhythm works in tandem with homeostatic pressures to regulate sleep and wakefulness (8).

While societal demands such as work start times may dictate sleep schedules, people exhibit diurnal preferences known as chronotypes that better reflect when they are most inclined to engage in sleep or activity if not restricted by external demands. Individuals can be classified as morning-type, intermediate-type, or evening-type depending on what time of day they would prefer to be most active. Interestingly, data from family and twin studies indicate that the heritability of chronotype is around 50% implying that this preference is not simply a product of environmental causes (9). An individual's chronotype often changes over their lifetime with children most likely to be morning chronotypes, adolescents exhibiting progressively later chronotypes until around age 20 years, and older adults primarily exhibiting morning diurnal preference (10). That said, there is still evidence for a near-normal distribution of chronotypes in all age groups suggesting that the evening chronotype does exist in older adulthood (10).

There are significant differences in health behaviors and health status between morning and evening chronotypes of all adult ages, including older adults. Evening chronotypes have been shown to spend more time resting and napping during the day (11), be more likely to experience disturbed sleep due to varying sleep schedules and accumulated sleep debt during the week (12), and consume fewer and larger meals during the day (13). Evening chronotypes also tend to consume more alcohol (14), are more likely to smoke, and engage in less physical activity compared to morning chronotypes (12). Perhaps as a result of these health behaviors, evening chronotypes exhibit a higher risk of hypertension (11) and are more likely to have type II diabetes and cardiovascular disease (15). In addition to physical health, evening chronotypes are more likely to use maladaptive emotion regulation strategies (16), which could partially explain why they are also more likely to be diagnosed with a psychiatric disorder compared to morning chronotypes (15). Of note, many studies that investigate relationships between chronotype and health behaviors or health status employ cross-sectional designs, so causal direction has not been definitively determined.

Less is known about the relationship between chronotype and cognitive performance. Some research (17) suggests that evening chronotypes perform better on cognitive tasks (specifically measures of memory, processing speed, working memory, and general cognitive ability). Preckel et al. (17) suggested that evening chronotypes may develop superior problem-solving capabilities due to a more frequent need to overcome challenges in everyday life brought about through a mismatch of diurnal preference and societal demands. Certainly, research suggests a differential impact of testing time on cognitive performance between chronotypes. Several studies suggest that evening chronotypes perform worse on tests of vigilance and executive function when tested in the morning compared to morning chronotypes (18). More studies have investigated the impact of chronotype on academic performance in children, adolescents, or young adults. Research in this vein indicates that eveningness is negatively associated with academic achievement, whereas morningness shows the opposite pattern (17) High school students with early chronotypes earn significantly higher grades than later chronotypes when tested in early and late morning hours, but this difference disappears if tested in the early afternoon (8). Researchers theorize that this can be attributed to a *synchrony effect* or the tendency for individuals to perform best at times of day that match their individual diurnal preference (17). Relatedly, research has also suggested that it is not chronotype itself but sleep disturbance and excessive daytime sleepiness that account for these differences in academic achievements (19).

Of note, the majority of these studies focus on younger populations, and less is known about the relationship between chronotype and cognitive performance in older adults. Using a dementia screening tool (the Mini-Mental Status Exam; MMSE) in a cognitively normal sample, Thapa and colleagues (20) found no significant difference in cognitive performance between older adult morning chronotypes and evening chronotypes, although it is worth noting that they did not measure or control for the time of day at which participants completed cognitive testing. In their older adult sample, McHugh, Walsh, and Lawlor (21) found that both morningness and eveningness behavior (determined by bedtime and rise time) was associated with poorer performance on cognitive measures (i.e., MMSE and tests from the Cambridge Cognition Examination) compared to individuals who adopted an average bedtime and rise time. This led authors to argue that either extreme serves as a marker of poor cognitive health. Taken together, findings from these two studies suggest that a relationship between cognitive performance and chronotype may exist in older adulthood and differs from that seen in younger populations. It is important not to generalize too freely from a limited number of studies and use of dementia screening tools. A major limitation of dementia screening tools is the associated ceiling effects (i.e., there is minimal variability in scores among individuals without cognitive impairment). At this time, the relationship between cognitive performance and chronotype in older adults requires further exploration.

It is unclear how often chronotype is considered when designing interventions to increase engagement in physical activity. However, the biopsychosocial model suggests that it may be useful to take this into account. This model, which advocates for a holistic approach to the concept of health and disease, suggests that factors related to an individual's biological makeup, psychology, and social and physical environments all contribute and interact to influence health (22, 23). Therefore, in the case of cognitive performance, it is wise to consider the role of a biologically determined chronotype when scheduling exercise sessions because this could influence motivation to engage in activity at the scheduled start-time. For example, if an older adult with an evening chronotype prefers to be active in the evening but does not feel safe walking in their neighborhood at night, this may discourage any engagement in activity, which is not apparent in someone living in the same neighborhood with a morning chronotype. This idea is supported by Didikoglu and colleagues (11) who found that the number of reported difficulties getting around (e.g., going outdoors) was higher among those reporting evening chronotype compared to morning chronotype. If older adults with evening chronotypes are actually engaging in less physical activity, it would be worth exploring why, and then adjusting the timing of exercise interventions to account for all chronotypes (e.g., offering more options).

In addition to influencing motivation to engage in activity, a participant's chronotype could determine how physical activity affects their body. For example, when high-intensity interval exercise is performed in the morning, it is more physically stressful for evening chronotypes as evidenced by a slower return to baseline levels of cortisol following exercise termination (6). Morning exercise stimulates physiologically different processes than evening exercise, and if a participant is free to engage in a prescribed exercise intervention at the timing of their choice, the activity could impact them differently than someone who engages in the same activity at a non-preferred time. Chronotype may predict the ideal time of day to engage in exercise to achieve maximal performance, and researchers have determined that morning chronotypes exhibit peak performance 5.5 h after waking whereas evening chronotypes perform best 11 h after waking (24). Interestingly, the time of day at which exercise is performed has been shown to moderate the relationship between chronotype and exercise such that morning exercise increases exercise frequency in people with morning chronotypes but decreases it in people with evening chronotypes (25). It is possible that this is related to the degree of perceived exercise exertion as morning chronotypes perceive less exertion when engaging in morning activity compared to evening chronotypes (26).

The most reliable measure of circadian timing is the dim light melatonin onset [DLMO (27)]. While highly accurate, collection of the DLMO is costly and time-consuming and places a heavy burden on both participants and research staff. For this reason, chronotype is most commonly assessed through self-report. Researchers often use questionnaires such as the Morningness-Eveningness Questionnaire [MEQ (28)] and the Munich Chronotype Questionnaire [MCTQ (29)]. The MEQ measures psychological preference for day-time and sleep behavior, and it is the most widely used chronotype assessment tool (30). In contrast, the MCTQ focuses on sleep timing, and it uses the midpoint of sleep on work-free days to estimate chronotype on a dimension ranging from eveningness to morningness (29). The MCTQ is considered more objective than the MEQ since participants are asked to report their bedtimes and wake times, and it also accounts for potential differences in sleep behavior on workdays and free days (30).

Researchers have recently argued that body-worn actigraphy devices can objectively measure chronotype (31). These unobtrusive devices can capture information regarding the wearer's bedtime, sleep duration, and arise time, and the midpoint of the sleep interval can be used as a proxy for chronotype since this avoids any influence of sleep duration. Actigraphy-calculated midpoints of sleep significantly correlate with MCTQ midpoints, and the differences between measures do not seem to vary by chronotype (32). Despite actigraphy's utility in measuring this construct, it appears more common to use actigraphy alongside standard questionnaires to estimate chronotype [e.g. (18, 33),]. In our study, we used actigraphy to assess chronotype without a supplemental self-report measure, which offered a relatively novel means of investigating this construct that otherwise relies solely on the manifestation of an inherently subjective characteristic. More similar to the MCTQ, it measures behaviors rather than self-reported preferences. To better reflect the categorical approach to conceptualizing chronotype that is typical of self-report measures, we employed a sample-dependent classification system to group participants into morning, intermediate, or evening chronotype. To our knowledge, there is no standardized set of criteria using sleep midpoints to accomplish this task, and so this was also a novel approach to exploring this concept. Similar to chronotype, physical activity can be assessed objectively *via* actigraphy, reducing biases associated with self-reported recall. This approach captures participants' movement in a free-living environment over an extended period of time (e.g., 24 h a day for a full 7 days).

Cognition can be operationalized in myriad ways such as the presence or absence of a medical diagnosis (e.g., Alzheimer's disease), performance on a brief dementia screening tool (e.g., MMSE), or performance on an extended neuropsychological battery. While dementia screening tools are useful for identifying areas of concern, they are limited in the information they provide for participants who are cognitively normal and are subject to floor and ceiling effects. Despite this fact, dementia screening tools are frequently used in research to represent cognitive function in cognitively normal older adults. In contrast, more detailed neuropsychological assessments designed to test a range of cognitive ability levels enable quantification of participants' cognitive strengths and weaknesses in several cognitive domains (e.g., memory, attention, executive function, language, visuospatial skills), which provides a richer source of information when cognition is the outcome of interest. Thus, results of a neuropsychological battery will be utilized in this study.

Despite there being a known association between chronotype and sleep disruption (12), the literature is inconsistent when it comes to managing the potential influence of diagnosed sleep disorders on findings. Some researchers intentionally exclude participants with sleep disorders, others control for their effects in analyses, and others do not explicitly mention sleep disorders. Disturbed sleep is a common experience for older adults, with anywhere from one to two-thirds reporting sleep complaints, depending on the number of comorbid conditions experienced (34, 35). Variables related to disturbed sleep (i.e., sleep duration and excessive daytime sleepiness) are associated with worse cognitive performance (36) as are variables related to structured physical activity (3). For this reason, true relationships between cognitive performance, physical activity, and chronotype are difficult to decipher when sleep disorders are not part of the research design or statistical analyses. We attempted to address this by analyzing data both including and excluding the data of participants with self-reported sleep disorders.

The existing literature on chronotype, physical activity, and cognition has largely overlooked older adults, relied on dementia screening tools to assess cognition in cognitively normal adults, and used self-report instruments to measure chronotype and physical activity. Furthermore, the inclusion or exclusion of individuals with sleep disorders has been inconsistent.

The present study aimed to expand on existing literature through use of objective means to measure chronotype and physical activity and an extensive neuropsychological battery to measure cognition in a sample consisting of participants with and without sleep disorders and again in the same sample excluding participants with sleep disorders. We hypothesized that (1) evening-type older adults would engage in lower levels of total and peak physical activity compared to older adults with an intermediate or morning-type chronotype. We also expected to find that activity patterns across the day varied by chronotype such that evening-types were more active later in the day than morning-types and vice versa. Because older adults with an evening chronotype exhibit worse health behaviors and health status, we predicted (2) that evening-types would exhibit worse cognitive performance compared to individuals with intermediate or morning-type chronotype. Finally, we (3) refrained from generating specific predictions regarding any changes in outcome when participants with sleep disorders were included or excluded from analyses. We believed their exclusion may eliminate relationships between chronotype and outcomes of interest if disordered sleep is the driving factor behind findings from the literature. However, if it is truly sleep timing and differences in diurnal patterns of activity that explain relationships between chronotype and health outcomes, we believed that excluding participants with sleep disorders would not alter our findings. We considered this final study aim an exploratory part of our analyses.

## Materials and methods

### Participants

From 2015 to 2022, participants were recruited from the University of Kansas Alzheimer's Disease Research Center (KU-ADRC) Clinical Cohort for enrollment in a sub-study measuring physical activity and sleep using wrist-worn accelerometry. The Clinical Cohort is an ongoing longitudinal study of greater than 1,000 participants. It annually collects demographic, medical, psychological, and cognitive data with the goal of developing and maintaining a well-characterized group of study participants with and without cognitive impairment due to Alzheimer's disease. To be included in the KU-ADRC Clinical Cohort, participants must be at least 60 years old and cannot exhibit significant depressive symptoms. Participants are also excluded if they have untreated thyroid dysfunction that could negatively impact cognitive performance or if they have any systemic illness that could prevent completion of a required annual evaluation. All participants who meet inclusion requirements for the Clinical Cohort are eligible to participate in the sub-study that is ongoing and aims to collect longitudinal data from its current participants.

The present analysis of the sub-study excluded participants with cognitive impairment at the time of data collection determined by a clinical dementia rating (CDR) score of >0 and if they used a device to assist with ambulation. We did not exclude participants with self-reported sleep disorders (i.e., sleep apnea, REM sleep behavior disorder, hyposomnia, insomnia) or reported use of a pharmacologic sleep aid (i.e., benzodiazepines, barbiturates, and miscellaneous anxiolytics, sedatives, and hypnotics). We evaluated the results of each analysis with and without these participants included.

### Procedure

Beginning in 2015, research coordinators approached participants every other year at their regularly scheduled annual clinical cohort evaluation to recruit them for participation into an observational sub-study of physical activity and sleep using accelerometry. When providing informed consent, participants were told that they would be asked to wear a GT9X link accelerometer on their non-dominant wrist for 7 days while continuing to move freely in their environment and go about their regular activities.

Participants were fitted with a watchband containing the GT9X that was programmed to begin recording activity at the start of their scheduled study visit. Participants were provided with verbal and written instructions on how to wear the watch and were asked to keep the watch fitted snuggly to their non-dominant wrist at all times for a full 7 days and not remove it even when bathing or sleeping. Watches were programmed to display the time of day enabling participants to use it as a wristwatch.

Participants were asked to keep a nightly sleep log. Written instructions directed participants to record the time and length of any naps they took, the time they got into bed, the time they fell asleep, how long it took to fall asleep, the time they awoke in the morning, the time they got out of bed in the morning, and any additional information that could impact sleep (e.g., travel, illness). Research staff provided prepaid padded envelopes in which participants returned the sleep logs and GT9X.

Typically, on the same day of their annual study visit, participants completed a cognitive test battery. For those unable to schedule these sessions consecutively, every effort was made to ensure that study participants completed the cognitive test battery as close to their clinical cohort visit as possible. The cognitive test battery was administered by trained research staff who are routinely audited to ensure close attention to standardization. The cognitive assessment typically required 1.5 h to complete.

At the annual study visit, research staff collected participants' height and weight, which was used to calculate their body mass index (BMI). Participants reported their date of birth, sex, and educational attainment upon initial enrollment into the KU-ADRC Clinical Cohort. Because of their known association with cognition, sleep, and physical activity, these variables were included as covariates in analyses. To account for differences in optimal performance based on testing time, the discrepancy between chronotype and time of cognitive testing was also entered as a covariate in models predicting cognitive performance. Informed consent forms and all study procedures were approved by the KU-ADRC's Institutional Review Board.

### Measures

#### Actigraphy

We used GT9X Link accelerometers (Pensacola, FL) to measure total physical activity, peak physical activity, and chronotype. The GT9X devices were programmed to collect data at a sample rate of 30 Hz. Raw data were downloaded and processed using ActiLife software version 6.13.2 or 6.13.4 (ActiGraph, LLC) and re-integrated into 60-second epochs. Optional settings such as the low frequency extension (LFE) filter and inclinometer were disabled, as they have been shown to alter estimates in physical activity outcomes differentially in more and less active participants (37). The Cole-Kripke algorithm (38) was used to process sleep data according to a standardized protocol. For each night of data, ActiGraph-detected sleep intervals were compared to participants' sleep logs to determine the sleep interval. If the sleep log closely matched the ActiGraph-detected sleep interval (i.e., the time in bed and time out of bed from the sleep log were within 30 min of their counterparts determined by ActiLife software), the ActiGraph-detected sleep interval was retained. If there was more than a 30-minute difference between the sleep log and ActiGraph-detected sleep intervals, the researcher manually scored the sleep data in ActiLife by visually inspecting sharp increases or decreases in bouts of activity. This final step was automatically employed when participants failed to complete or return their sleep logs.

Wear time was validated using the algorithm developed by Choi, Liu, Matthews, and Buchowski (39). A valid day of wear required a minimum of 10 h of wear time, participants needed to have at least one weekend day, and participants with less than 4 days of valid data were excluded from analyses.

#### Physical activity

The GT9X captures raw acceleration across three orthogonal planes (vertical, medio-lateral, and antero-posterior). The ActiLife™ software can be used to calculate the vector magnitude (VM), a composite score of activity counts across all three axes. The Montoye cut points (40) were used to classify activity by intensity level. According to these cut points, <2,860 VM counts per minute (CPM) is classified as sedentary, 2,860–3,940 as light, and >3,941 as moderate to vigorous. We acknowledge that these cut-points have limitations as they have not been validated in an older adult sample and they use a sedentary label without collecting information on a wearer's posture. However, they were developed for use in wrist-worn data, and so they are better suited to classify activity in our participants compared to cut-points developed for hip-worn devices [e.g. (41),]. These cut points were used solely to facilitate descriptive analyses and did not serve as the primary measure of physical activity.

Other commonly used physical activity outcomes (e.g., total step counts) are not reliably estimated by the wrist-worn GT9X, and arbitrarily categorizing physical activity with cut points limits interpretation and analysis. Therefore, the average VM CPM (averaged across the week) served as our primary measure of total physical activity. This continuous variable reflects total activity throughout the day.

We estimated peak activity (greatest VM CPM reached in a day) and the average time of day at which this occurred as parallel estimates to the sleep midpoint to allow comparison of peak activity times given differences in chronotype. Furthermore, we calculated average VM CPM within six 4-hour intervals across a 24-hour day (6:01am – 10:00am, 10:01am – 14:00pm, 14:01pm – 18:00pm, 18:01pm – 22:00pm, 22:01pm – 2:00am, and 2:01am – 6:00am) to enable analyses investigating differences in diurnal activity patterns among chronotypes.

#### Chronotype

This study employed a novel approach for measuring chronotype through actigraphy. The GT9X captures information regarding the wearer's bedtime and arise time, and it can be used to measure the median point of the sleep interval. This midpoint (averaged across all valid nights of wear) captured by the GT9X was used to represent chronotype (31, 32).

To allow comparison to previous published literature, we calculated a categorical chronotype variable *via* a sample-dependent classification system. We calculated the mean and standard deviation of the sleep midpoint in participants without a reported sleep disorder. Using this, all individuals (including those with sleep disorders) were categorized as “morning-types” (sleep midpoint >1 SD below the sample mean midpoint), “intermediate-types” (sleep midpoint within 1 SD of the sample mean midpoint), or “evening-types” (sleep midpoint >1 SD above the sample mean midpoint).

#### Cognitive performance

As part of their annual evaluation, participants complete a cognitive test battery that is part of the National Alzheimer's Coordinating Center (NACC) Uniform Data Set (UDS), with additional tests added. The final test battery included in our analyses consist of one subtest from the Wechsler Adult Intelligence Scales-Revised [WAIS-R; Digit Symbol Substitution Test (42);], two subtests from the Wechsler Memory Scales-Revised [WMS-R; Letter Number Sequencing and Digit Span Forward and Backward (43);], the Stroop Test [interference condition (44);], the Craft Story 21 Immediate and Delayed recall (45), the Free and Cued Selective Reminding Test (46), two tests of semantic verbal fluency [animal and vegetable naming (47);], and Trail Making Test B (48). Tests in this battery are thought to assess working memory, auditory memory, episodic memory, processing speed, attention, cognitive flexibility and executive control, language, and visuospatial skills (49–52). Cognitive tests in the UDS were selected under the guidance and approval of a Clinical Task Force assembled by the National Institute on Aging, and normative test scores are available to its users (53). Cognitive batteries like the UDS far outperform dementia screening tools in their diagnostic utility (54).

Rather than raw or standardized scores from individual cognitive tests, the present study used cognitive factor scores derived from a confirmatory factor analysis described elsewhere [e.g. (55),]. These factor scores represented verbal memory (immediate and delayed logical memory, selective reminding test trials sum), attention (digits forward, digits backward, letter-number sequencing), and executive function (category fluency sum of animals and vegetables, Stroop color word interference, Trail Making Test B, and digit symbol substitution test). These factor scores served as this study's measure of cognitive performance.

### Design and analysis

To explore differences in diurnal physical activity patterns and cognitive performance among chronotypes, we used multivariate analysis of covariance (MANCOVA). First, we modeled the relationship between total VM CPM, peak activity, and time of peak activity and the main predictor of interest, chronotype group (evening-type vs. intermediate-type vs. morning-type) controlling for age, sex, education, and BMI. Second, we modeled the relationship between average VM CPM for each of the six 4-hour time intervals (dependent variables) and chronotype group, controlling for the aforementioned covariates. Finally, we modeled the relationship between verbal memory, attention, and executive function (dependent variables) and chronotype group, controlling for covariates including the discrepancy between sleep midpoint and time of cognitive testing as well as VM CPM. To explore whether findings changed when participants with sleep disorders were excluded from analyses, we ran all three aforementioned models a second time in participants without a sleep disorder. All statistical analyses were conducted with SPSS version 24 for Mac (SPSS, Inc.; Chicago, IL). The alpha level was set *a priori* at.05. There were no missing data.

## Results

### Descriptive statistics

#### Sample demographics

Of the 210 participants with available data, 57 were omitted based on exclusionary criteria. This included having a CDR score >0 (*n* = 52) and using a device to assist with ambulation (*n* = 5). This left a final sample of 153 participants. Observed power ranged from .36 to .99 for primary analyses and from .45 to .99 for exploratory analyses.

The final sample was 61.44% female, 89.54% right-hand dominant, 100% English-speaking, and 78.43% no longer employed full time. Additionally, 28.76% had one or more reported sleep disorder diagnosis. Those with a sleep disorder reported significantly higher BMIs (*M *= 29.80, *SD *= 6.02) than those without (*M *= 26.97, *SD *= 3.66), *t* (56.28) = −2.91, *p *= .005. No other demographic characteristic differed between participants with and without a sleep disorder. See Table 1 for additional participant demographic characteristics.

**Table 1:** Descriptive statistics.

Participant characteristics	Men (*n* = 59)		Women (*n *= 94)		Total (*N *= 153)	
	M	SD	M	SD	M	SD
Age (years)*	71.98	5.73	69.32	5.78	70.35	5.89
Education (years)	16.98	2.87	16.22	2.84	16.52	2.87
BMI	28.36	4.59	27.42	4.63	27.78	4.62
	*N*	%	*N*	%	*N*	%
**Race**						
White	56	94.92	85	90.43	141	92.16
Black/African American	2	3.39	7	7.45	9	5.88
Asian American	1	1.69	2	2.13	3	1.96
Hispanic/Latino Ethnicity	0	0.00	1	1.06	1	0.65
**Marital Status** *						
Married	54	91.53	56	59.57	110	71.90
Widowed	1	1.69	9	9.57	10	6.54
Divorced	2	3.39	20	21.28	22	14.38
Separated	0	0.00	1	1.06	1	0.65
Never Married	2	3.39	4	4.26	6	3.92
Living as Married	0	0.00	2	2.13	2	1.31
**Living Situation** *						
Spouse/Partner	52	88.14	58	61.70	110	71.90
Alone	5	8.47	31	32.98	36	23.53
Relative/Friend	1	1.69	2	2.13	3	1.96
Group	0	0.00	2	2.13	2	1.31
**Sleep Disorder**						
Sleep Apnea	12	20.34	16	17.02	28	18.30
RBD	1	1.69	1	1.06	2	1.31
Insomnia	0	0.00	2	2.13	2	1.31
Hyposomnia	3	5.08	12	12.77	15	9.80

* Men and women differ at *p* < .05. BMI, body mass index; RBD, rapid eye movement sleep behavior disorder.

#### Cognitive testing

Fifty-eight participants completed cognitive testing in the morning (with start times between 08:00 and noon) and 95 completed it in the afternoon (with start times between noon and 15:30). The average discrepancy between the sleep midpoint and time of cognitive testing was 9.06 h (*SD* = 2.33 h) and ranged from 3.40 to 19.14 h. This discrepancy is included in analyses to account for differences between individuals in match between preferred times of day and actual testing times. The average length of time between cognitive testing and actigraphy data collection was 13.20 days (*SD* = 29.29 days) and ranged from 0 to 190 days.

#### Physical activity

**Figure 1:**
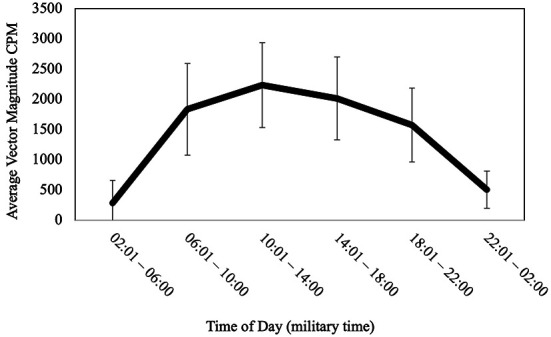
Activity patterns across the 24-hour cycle.

On average, participants engaged in 1,937.98 total VM CPM (*SD* = 559.14), which indicates overall low levels of activity compared to CDC recommendations for healthy levels of activity in older adults. Participants spent most of their average daily waking time in sedentary behavior (*M *= 75.93%, *SD* = 9.08) and less time in light physical activity (*M* = 18.89%, *SD* = 6.40%) and MVPA (*M* = 5.18%, *SD* = 4.29%). Average peak physical activity (time of day when the highest intensity was achieved defined by VM CPM) was 11,662.15 (*SD* = 2,672.36) and occurred at 12:48:26. See Figure 1 for a visual representation of activity patterns across the 24-hour cycle for all participants.

#### Sleep characteristics

**Figure 2:**
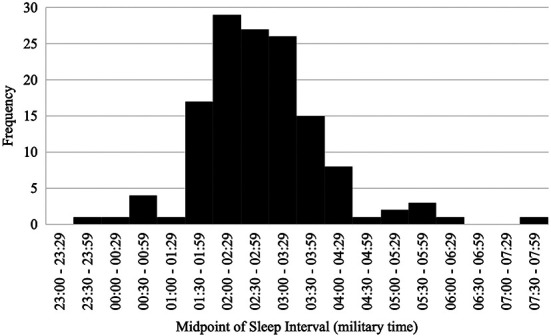
Distribution of continuous chronotype.

On average, participants went to bed at 22:54:39.69 (*SD* = 01:09:42.56), fell asleep at 22:57:38.04 (*SD *= 1:09:44.32), and arose in the morning at 06:48:35.92 (*SD* = 01:14:14.99). Participants slept for an average of 409.52 min (*SD* = 62.40; *M* = 6.83 h, *SD* = 1.04 h) and the duration of wake after sleep onset (WASO) was 61.32 min (*SD* = 30.79). Average sleep efficiency was 86.57% (*SD* = 6.23%). The average midpoint of the sleep interval for the entire sample was 02:51:37 (*SD* = 01:04:53.87) and ranged from 23:32:00 to 07:42:30 (see Figure 2). None of the aforementioned sleep variables significantly differed between participants with and without a reported sleep disorder, all *p*s* *≥ .194.

Morning-types (i.e., individuals with a sleep midpoint <1 SD below the sample mean midpoint; *n* = 14) had an average sleep midpoint of 01:05:46 (*SD* = 0:40:12.43), intermediate-types (i.e., individuals with a sleep midpoint within 1 SD of the sample mean; *n* = 121) had an average sleep midpoint of 02:46:08 (*SD* = 00:33:55.20), and evening-types (i.e., individuals with a sleep midpoint >1 SD above the sample mean; *n* = 18) had an average sleep midpoint of 04:50:49 (*SD* = 01:01:45.00). Total sleep time, WASO, and sleep efficiency did not significantly differ between chronotypes, all *p*s* *≥ .56.

The demographic characteristics sex, race, marital status, living situation, and handedness did not significantly differ between chronotype groups, all *p*s ≥ .415. Similarly, presence of self-reported sleep apnea, RBD, insomnia, and hyposomnia did not significantly differ between groups, all *p*s ≥ .126. Covariates age and years of education did not significantly differ between chronotype groups, all *p*s ≥ .58. However, covariate BMI significantly differed such that evening-types had greater BMIs than morning-types, *p *= .01. Additionally, the discrepancy between sleep midpoint and cognitive testing time significantly differed between chronotype groups such that morning-types had higher discrepancies than both intermediate-types (*p *= .04) and evening-types (*p *= .001). This suggests that morning-types may have more frequently engaged in cognitive testing at a less-preferred time of day. Cognitive testing times did not significantly differ between chronotypes, *F* (2, 150) = 1.297, *p *= .276, *η*^2 ^= .02.

### Primary analyses

#### Chronotype predicting total VM CPM, peak activity, and time of peak activity

Table 2 presents findings from the MANCOVA model predicting physical activity (total VM CPM) and diurnal patterns of behavior (average peak activity and time of peak activity) by chronotype, controlling for covariates (age, sex, education, and BMI). Total VM CPM did not significantly differ between chronotype groups, *F* (2, 146) = 2.18, *p *= .117, partial *η*^2 ^= .03 (observed power = .44). Similarly, average peak activity did not significantly differ between chronotype groups, *F* (2, 146) = 1.71, *p *= .185, partial *η*^2 ^= .02 (observed power = .36).

**Table 2:** Chronotype group differences in total activity, peak activity, and time of peak activity (*N *= 153).

	Morning-type (*n* = 14)		Intermediate-type (*n* = 121)		Evening-types (*n* = 18)			
Source	M	SE	M	SE	M	SE	F (2, 146)	*p*
Total VM CPM	2,055.99	139.06	1,958.64	46.61	1,707.31	122.70	2.18	.117
Average Peak Activity	12,390.92	712.29	11,722.46	238.74	10,689.87	628.49	1.71	.185
Time of Peak Activity	10:50:13	0:44:51	12:56:09	0:15:02	13:28:37	0:39:35	4.05	.019

MANCOVA model predicting total VM CPM, average peak activity, and time of peak activity from chronotype, controlling for covariates (age, sex, education, and BMI). VM CPM, vector magnitude counts per minute; BMI, body mass index.

**Figure 3:**
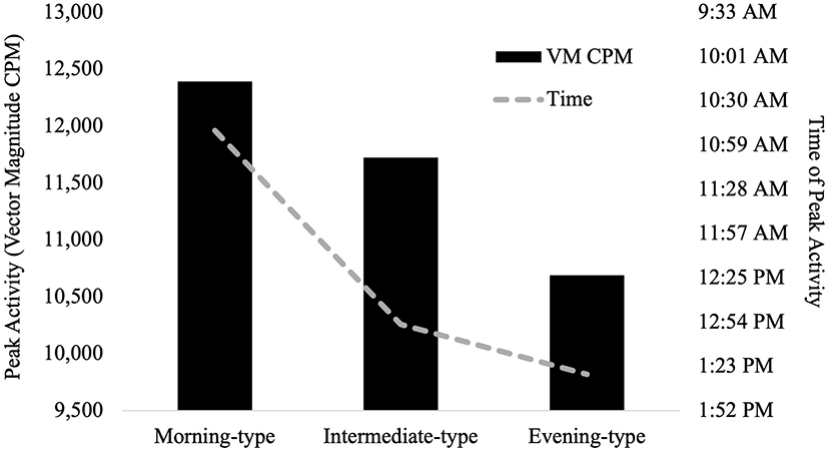
Differences in average peak activity and time of peak activity between chronotype groups.

Time of peak activity significantly differed between chronotype groups, *F* (2, 146) = 4.05, *p *= .019, partial *η*^2 ^= .05 (observed power = .71). Pairwise comparisons indicated that morning-types engaged in peak activity significantly earlier than both intermediate-types (*p *= .009), and evening-types (*p *= .01). The time of peak activity did not significantly differ between intermediate-types and evening-types, *p *= .445. See Figure 3.

#### Chronotype predicting average VM CPM across 24-hour cycle

**Figure 4:**
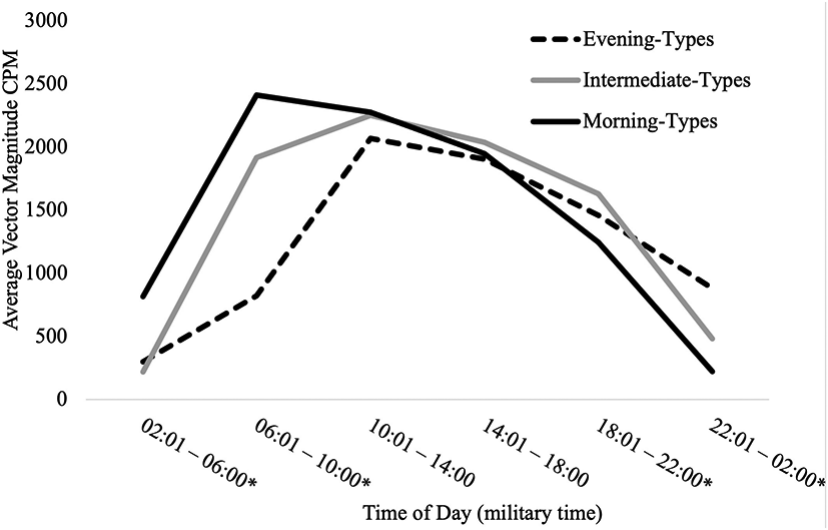
24-hour activity patterns of chronotype groups. *=at least one significant difference between chronotype groups at *p *< .05. Within the interval of 2:01–6:00, morning-types engaged in significantly more activity than intermediate-types (*p* < .001) and evening-types (*p* < .001). Activity exhibited by intermediate-types and evening-types during this interval did not differ (*p* = .356). Within the interval of 6:01–10:00, morning-types engaged in significantly more activity than intermediate-types (*p* = .006) and evening-types (*p* = .006). Intermediate-types also engaged in significantly more activity than evening-types (*p* < .001). Within the interval of 18:01–22:00, Intermediate-types engaged in significantly more activity than morning-types (*p* = .021) but not evening-types (*p* = .256). Activity exhibited by morning-types and evening-types during this interval did not differ (*p* = .311). Within the interval of 22:01–2:00, evening-types engaged in significantly more activity than morning-types (*p* < .001) and intermediate-types (*p* < .001). Intermediate-types also engaged in significantly more activity than morning-types (*p* < .001).

Table 3 presents findings from the MANCOVA model predicting average total VM CPM within six 4-hour time intervals across a 24-hour day (2:01–6:00, 6:01–10:00, 10:01–14:00, 14:01pm – 18:00, 18:01–22:00, and 22:01–2:00) by chronotype, controlling for covariates (age, sex, education, and BMI). Average total VM CPM occurring during the intervals of 6:01–10:00, 18:01–22:00, 22:01–2:00, and 2:01–6:00 significantly differed between chronotype groups, all *p*s < .001, all partial *η*^2 ^≥ .21 (all observed power ≥.99). See Figure 4 for a visual depiction of the 24-hour activity patterns of each chronotype group.

**Table 3:** Chronotype group differences in total activity within six 4-hour intervals (*N *= 153).

VM CPM	Morning-type (*n* = 14)		Intermediate-type (*n* = 121)		Evening-types (*n* = 18)				
Source	M	SE	M	SE	M	SE	F (2, 146)	*p*	Partial *η*^2^
02:01–06:00	814.48	91.53	220.28	30.68	300.41	80.76	19.49	<.001	.21
06:01–10:00	2,411.17	167.65	1,915.86	56.19	822.62	147.92	29.70	<.001	.29
10:01–14:00	2,275.29	180.99	2,251.98	60.66	2,067.82	159.70	0.60	.548	.01
14:01–18:00	1,946.99	176.87	2,036.61	59.28	1,903.31	156.07	0.40	.669	.01
18:01–22:00	1,247.11	155.38	1,628.30	52.08	1,460.79	137.10	3.15	.046	.04
22:01–02:00	223.69	71.32	482.85	23.90	880.71	62.93	25.38	<.001	.26

MANCOVA model predicting average total VM CPM within six 4-hour time intervals from chronotype, controlling for covariates (age, sex, education, and BMI). VM CPM, vector magnitude counts per minute; BMI, body mass index.

#### Chronotype predicting verbal memory, attention, and executive function

Table 4 presents findings from the MANCOVA model predicting verbal memory, attention, and executive function factor scores by chronotype, controlling for covariates (age, sex, education, BMI, the discrepancy between sleep midpoint and time of cognitive testing, and VM CPM). Verbal memory scores (*p* = .978, partial *η*^2 ^< .001, observed power = .05) did not significantly differ between chronotype groups. Attention scores significantly differed between chronotype groups, *p *= .042, partial *η*^2 ^= .04, (observed power = .61). Pairwise comparisons showed that evening-types exhibited significantly worse attention than intermediate-types (*p *= .012) but not morning-types (*p *= .067). There was no difference in scores between intermediate-types and morning-types (*p *= .874). A similar pattern of results was evident in the cognitive domain of executive function in that scores significantly differed between chronotype groups, *p* = .011, partial *η*^2 ^= .06 (observed power = .78). Pairwise comparisons indicated that evening-types exhibited significantly worse executive function performance than intermediate-types (*p *= .014) but not morning-types (*p *= .750). There were no differences in scores between intermediate-types and morning-types (*p *= .068).

**Table 4:** Chronotype group differences in verbal memory, attention, and executive function factor scores (*N *= 153).

	Morning-type (*n* = 14)		Intermediate-type (*n* = 121)		Evening-types (*n* = 18)			
Source	M	SE	M	SE	M	SE	F (2, 144)	*p*
Verbal Memory	1.04	0.25	1.05	0.08	1.10	0.22	0.02	.978
Attention	0.32	0.10	0.30	0.03	0.07	0.09	3.25	.042
Executive Function	0.37	0.14	0.63	0.04	0.31	0.12	4.68	.011

MANCOVA model predicting verbal memory, attention, and executive function factor scores from chronotype, controlling for covariates (age, sex, education, BMI, discrepancy between sleep midpoint and time of cognitive testing, and VM CPM). VM CPM, vector magnitude counts per minute; BMI, body mass index.

#### Post hoc exploratory analyses

We chose not to exclude participants with reported sleep disorders from our analyses to allow exploration of their role in driving the relationship between sleep timing, physical activity, and cognitive performance. As noted above, sleep characteristics did not differ between participants with and without a reported sleep disorder. Additionally, our primary variables of interest (i.e., VM CPM, peak activity, time of peak activity, VM CPM within six 4-hour intervals, and performance across three cognitive domains) did not significantly differ between those with and without a sleep disorder, all *p*s ≥ .284. Therefore, we chose to include these participants to preserve power for our analyses.

As noted in the introduction, the decision whether to include or exclude participants with sleep disorders from studies investigating chronotype appears inconsistent across researchers. In an effort to better understand the unique influence of sleep timing over and above sleep dysregulation, we opted to re-run our primary analyses in the 109 participants without a reported sleep disorder. In particular, we wanted to examine whether our finding of evening-types exhibiting significantly worse executive function performance than intermediate-types would remain once participants with a sleep disorder were removed from analyses.

The 109 participants without a reported sleep disorder did not significantly differ from participants with sleep disorders in sex, age, education, racial makeup, marital status, or living situation (all *p*s ≥ .470). Participants with a sleep disorder had significantly higher BMIs than those without, *t* (151) = −3.55, *p *< .001.

#### Chronotype predicting total VM CPM, peak activity, and time of peak activity in participants without a sleep disorder

Table 5 presents findings from the MANCOVA model predicting physical activity (total VM CPM) and diurnal patterns of behavior (average peak activity and time of peak activity) by categorical chronotype, controlling for covariates (age, sex, education, and BMI). Total VM CPM significantly differed between chronotype groups, *F* (2, 102) = 4.38, *p *= .015, partial *η*^2 ^= .08 (observed power = .75). Pairwise comparisons indicated that evening-types engaged in significantly fewer total VM CPM than both morning-types (*p *= .01) and intermediate-types (*p* = .007). These findings differ from those seen in analyses when individuals with sleep disorders were included that showed no significant difference in VM CPM between chronotypes. Average peak activity did not significantly differ between chronotype groups, *F* (2, 102) = 2.24, *p *= .112, partial *η*^2 ^= .04 (observed power = .45), which is consistent with results from analyses including participants with sleep disorders.

**Table 5:** Chronotype group differences in total activity, peak activity, and time of peak activity (*N *= 109).

	Morning-type (*n* = 10)		Intermediate-type (*n* = 87)		Evening-types (*n* = 12)			
Source	M	SE	M	SE	M	SE	F (2, 102)	*p*
Total VM CPM	2,121.45	166.97	1,970.63	55.84	1,527.87	151.71	4.38	.015
Average Peak Activity	12,290.33	852.58	11,689.04	285.14	10,092.42	774.69	2.24	.112
Time of Peak Activity	10:30:19	0:54:20	13:06:52	0:18:10	13:58:42	0:49:22	4.51	.013

MANCOVA model run with sleep disorders excluded. Model predicts total VM CPM, average peak activity, and time of peak activity from chronotype, controlling for covariates (age, sex, education, and BMI). VM CPM, vector magnitude counts per minute; BMI, body mass index.

Time of peak activity significantly differed between chronotype groups, *F* (2, 102) = 4.51, *p *= .013, partial *η*^2 ^= .08 (observed power = .76). Pairwise comparisons indicated that morning-types engaged in peak activity significantly earlier than both intermediate-types (*p *= .007), and evening-types (*p *= .006). The time of peak activity did not significantly differ between intermediate-types and evening-types, *p *= .327. These findings are consistent with analyses that included participants with sleep disorders.

#### Chronotype predicting average VM CPM across 24-hour cycle in participants without a sleep disorder

Table 6 presents findings from the MANCOVA model predicting average total VM CPM within six 4-hour time intervals across a 24-hour day (2:01–6:00, 6:01–10:00, 10:01–14:00, 14:01pm – 18:00, 18:01–22:00, and 22:01–2:00) by chronotype category, controlling for covariates (age, sex, education, and BMI). Average total VM CPM occurring during the intervals of 6:01–10:00, 22:01–2:00, and 2:01–6:00 significantly differed between chronotypes categories, all *p*s < .001, all partial *η*^2 ^≥ .22 (all observed power ≥.99). These findings are consistent with those seen in analyses that included participants with a sleep disorder with the sole exception being that the interval 18:01–22:00 also differed between groups when the additional participants were included.

**Table 6:** Chronotype group differences in total activity within six 4-hour intervals (*N *= 109).

VM CPM	Morning-type (*n* = 10)		Intermediate-type (*n* = 87)		Evening-types (*n* = 12)				
Source	M	SE	M	SE	M	SE	F (2, 102)	*p*	Partial *η*^2^
02:01–06:00	984.42	118.45	204.84	39.61	308.17	107.63	19.49	<.001	.28
06:01–10:00	2,455.17	199.95	1,948.78	66.87	755.22	181.69	23.49	<.001	.32
10:01–14:00	2,201.16	227.48	2,289.69	76.08	1,843.44	206.70	2.07	.132	.04
14:01–18:00	1,998.10	217.02	2,052.63	72.58	1,706.77	197.20	1.36	.262	.03
18:01–22:00	1,319.17	186.33	1,637.06	62.32	1,287.30	169.31	2.91	.059	.05
22:01–02:00	257.76	85.44	469.42	28.57	846.46	77.634	14.28	<.001	.22

MANCOVA model run with sleep disorders excluded. Model predicts average total VM CPM within six 4-hour time intervals from chronotype, controlling for covariates (age, sex, education, and BMI). VM CPM, vector magnitude counts per minute; BMI, body mass index.

Within the interval of 6:01–10:00, morning-types engaged in significantly more activity than intermediate-types (*p* = .018) and evening-types (*p* < .001). Intermediate-types also engaged in significantly more activity than evening-types (*p* < .001).

Within the interval of 22:01–2:00, evening-types engaged in significantly more activity than morning-types (*p* < .001) and intermediate-types (*p* < .001). Intermediate-types also engaged in significantly more activity than morning-types (*p* = .021).

Within the interval of 2:01–6:00, morning-types engaged in significantly more activity than intermediate-types (*p* < .001) and evening-types (*p* < .001). Activity exhibited by intermediate-types and evening-types during this interval did not differ (*p* = .37).

#### Chronotype predicting verbal memory, attention, and executive function in participants without a sleep disorder

Table 7 presents findings from the MANCOVA model predicting verbal memory, attention, and executive function factor scores by chronotype, controlling for covariates (age, sex, education, BMI, the discrepancy between sleep midpoint and time of cognitive testing, and VM CPM). The omnibus test indicated that chronotype was not a significant predictor, *F* (3, 97) = 1.63, *p *= .14, partial *η*^2 ^= .05 (observed power = .62). This indicates that none of the cognitive domains significantly differed between chronotype groups. This finding differs from that seen in analyses including participants with a sleep disorder that found a significant difference between chronotype groups in the cognitive domain of executive function.

**Table 7:** Chronotype group differences in verbal memory, attention, and executive function factor scores (*N *= 109).

	Morning-type (*n* = 10)		Intermediate-type (*n* = 87)		Evening-types (*n* = 12)	
Source	M	SE	M	SE	M	SE
Verbal Memory	0.99	0.31	1.05	0.10	1.33	0.28
Attention	0.25	0.11	0.32	0.04	0.08	0.10
Executive Function	0.35	0.16	0.63	0.05	0.44	0.15

MANCOVA model run with sleep disorders excluded. Model predicts verbal memory, attention, and executive function factor scores from chronotype, controlling for covariates (age, sex, education, BMI, discrepancy between sleep midpoint and time of cognitive testing, and VM CPM). VM CPM, vector magnitude counts per minute; BMI, body mass index.

## Discussion

The purpose of this study was to explore differences between chronotypes in physical activity and cognitive performance in a sample of older adults. By using actigraphy to objectively measure physical activity and chronotype and an extensive neuropsychological battery to assess cognitive performance, we expanded on past research that relied on self-report questionnaires and dementia screening tools. Aging is the primary risk factor for cognitive decline, and health behaviors such as physical activity are known to reduce this risk. Because an individual's chronotype impacts the timing of physical activity and activity patterns, expanding our understanding of these relationships can better inform interventions that target these health behaviors in older adults.

### Chronotype and physical activity

Based on previous research, we predicted that evening-type individuals would engage in the lowest levels of total physical activity. While we found that activity patterns differed between the groups, total physical activity levels did not. Our results certainly confirmed that the time of peak activity differed between chronotype groups (morning-types engaged in peak activity earlier than both intermediate- and evening-types). Similarly, our findings demonstrated anticipated differences in activity patterns across the 24-hour cycle. Morning-types engaged in significantly more total physical activity than both intermediate- and evening-types in the early hours of the day, a time when exercise may yield a greater reduction in blood pressure and better prevent muscle loss compared to the evening (5). In contrast, evening-types were significantly more active than intermediate- and morning-types later in the day when exercise has been shown to have more benefit for building muscles and burning fat (5).

Our findings did not support our prediction that total and peak activity levels would differ between chronotypes. This is inconsistent with previous findings in younger [e.g. (12, 56),] as well as older adult samples [e.g. (20),]. Reasons for this inconsistency may stem from discrepancies in the way we defined both chronotype and physical activity. For example, Suh and colleagues used a self-report measure of chronotype (the MEQ) and categorized their participants into morning-, intermediate-, and evening-types rather than measuring chronotype with actigraphy-assessed sleep behavior. They also used a self-report measure of physical activity [the Seven-Day Physical Activity Recall (57)]; that requires participants to estimate the amount of MVPA in which they engaged based on personal recall. Self-report measures of physical activity are known to be subject to biased recall, confusion regarding ambiguous terms (e.g., physical activity, intensity), and social desirability to respond favorably (58), which can limit their accuracy. This could explain why no differences in physical activity were found between chronotypes in our sample that relied on objective measurement of both variables.

In an older adult sample, Thapa and colleagues (20) used the self-reported MEQ to measure chronotype and a wrist-worn accelerometer to measure physical activity. Instead of reporting total physical activity *via* VM CPM, they used the number of minutes spent in MVPA as their measure of physical activity. To determine whether it was our focus on total physical activity rather than activity at the highest intensity that led to these discordant findings, we re-ran this analysis using minutes spent in MVPA instead of VM CPM. Findings indicated no significant difference in MVPA between chronotype groups in our sample (see Table 8). This suggests that differences between our findings and those of other studies that also used accelerometry to measure physical activity are not due to discrepancies in how we defined this construct.

**Table 8:** Chronotype group differences in MVPA (*N *= 153).

	Morning-type (*n* = 14)		Intermediate-type (*n* = 121)		Evening-types (*n* = 18)	
Source	M	SE	M	SE	M	SE
MVPA	5.39	1.10	5.29	0.37	4.25	0.96

Table 8 presents adjusted means and standard errors for MVPA from the MANCOVA model predicting physical activity (MVPA) by chronotype, controlling for covariates (age, sex, education, and BMI). MVPA did not significantly differ between chronotype groups, *F* (2, 146) = 0.52, *p *= .595, partial *η*^2 ^= .01 (observed power = .14). MVPA, moderate to vigorous physical activity; BMI, body mass index.

As noted in our results, we created our chronotype categories using a sample-dependent classification system where participants who fell above or below 1 SD of the sample mean sleep interval midpoint were deemed evening- or morning-types respectively. This reflects participant behavior, but it may not reflect their preference, which is better captured by self-report measures like the MEQ (see Implications and Limitations sections for further discussion of this important difference). While chronotype remains normally distributed across the lifespan, the mean of the chronotype distribution is known to shift such that older adults have a chronotype distribution favoring morningness (10). Therefore, an evening-type older adult (defined by sample-specific classification strategies like ours) may have an earlier sleep interval midpoint than an evening-type younger adult. While we acknowledge the importance of staying cautious when drawing comparisons between our findings and those of researchers who used self-report measures of chronotype, our results suggest that observed behavior (regardless of preference) may not influence activity levels.

Interestingly, when we excluded participants with a sleep disorder from our analyses, we found that evening-types did engage in significantly fewer total VM CPM than both morning- and intermediate-types. However, the average peak activity did not differ between chronotype groups. It is possible that this is the true pattern of findings for individuals without a sleep disorder, and it is consistent with the literature showing lower levels of physical activity in evening-types (12). Our results suggest that total activity may be accumulated in a variety of patterns that may be useful information for design of tailored lifestyle interventions. For example, some individuals' activity patterns may reflect bursts of MVPA combined with otherwise sedentary behavior, whereas others may have bursts of MVPA combined with more light physical activity than sedentary behavior.

We cannot comment on comparisons between participants with and without a sleep disorder or the true pattern of findings in individuals with a sleep disorder due to our limited sample size (*n *= 44). Additionally, participants in our sample are not well-characterized in terms of severity or duration of disorder or degree of impairment, as our sole descriptor is a self-report of presence or absence of at least one diagnosed sleep disorder. Therefore, we are unable to further speculate given the present data available.

### Chronotype and cognitive performance

Based on findings of previous research [e.g. (20),] and a known association between eveningness behavior and health outcomes related to cognitive health (15), we predicted that evening-types would exhibit worse cognitive performance than other chronotypes. Our findings partially supported this hypothesis. We found that evening-types exhibited significantly worse executive function and attention performance than intermediate-types but not morning-types. Performance in the cognitive domain of verbal memory did not differ between groups. This is consistent with older adult samples that have shown worse cognitive functioning in individuals with lower MEQ scores [indicating greater eveningness (20);]. When drawing comparisons with this study, it is important to note a major difference in the way cognitive performance is defined. Thapa and colleagues relied solely on a dementia screening tool (the MMSE) to measure cognitive functioning, whereas our study used cognitive factor scores derived from a full neuropsychological battery. Cognitive screeners are limited by ceiling effects that prevent variability in scores above the mean. Thapa and colleagues also appear to have included participants with a greater range of cognitive performance than our sample as they only excluded those with “significantly reduced cognitive function” (p. 3). In contrast, we excluded all participants with a CDR > 0, indicating any form of cognitive impairment. It is possible that our choice to do so led to reduced variability in the cognitive performance of our sample.

Another factor that impacts our ability to make comparisons with the literature relates to methodological differences in conceptualizing chronotype. McHugh, Walsh, and Lawlor (21) used the self-reported Pittsburgh Sleep Quality Index (PSQI) to assess time to bed and time to rise and categorized participants as early-, normal-, and late sleepers. Using the MMSE and several tests from the Cambridge Cognition Examination, they found that both extremes (early- and late-sleepers) were associated with worse cognitive performance, which is somewhat inconsistent with our findings. This could indicate that sleep dysregulation (rather than sleep timing) drives the relationship between chronotype and cognitive performance. The study did not explicitly mention whether it included participants with sleep disorders. Researchers have demonstrated an increased risk of cognitive decline and Alzheimer's disease in individuals with sleep problems (i.e., short and long sleep duration, low sleep quality, circadian rhythm disruption, insomnia, obstructive sleep apnea) compared to those without sleep problems (59). When we excluded participants with a sleep disorder from analyses, we found no differences between chronotypes for any of the three cognitive domains, which supports the supposition that sleep dysregulation, not sleep timing is a key driver of this relationship.

Our prediction regarding chronotype and cognitive performance was informed by the awareness that eveningness is associated with worse health behaviors and health outcomes [e.g. (11, 12, 15),], which was evident in the BMI of our participants but not in any other outcome (e.g., sleep duration, WASO, and sleep efficiency did not significantly differ between chronotype groups). For a number of reasons (noted in our limitations section), it is possible that our sample consisted of an overall healthier group of older adults compared to the general population. This explanation is supported by our descriptive statistics demonstrating that the cognitive factor scores of our sub-sample were higher and less variable than the larger sample from which they were derived that included cognitively impaired individuals.

### Implications

Our findings demonstrated expected differences in activity patterns between chronotype groups, but no difference in total or peak physical activity. These findings are somewhat inconsistent with the literature that has used subjective measures of chronotype. Additionally, we found that evening-types exhibited significantly worse executive function and attention performance than intermediate-types, but this difference was not apparent when participants with a sleep disorder were excluded from analyses. This begs the question – does objectively-assessed chronotype have any meaningful relationship with health outcomes or behavior independent of sleep dysregulation?

Findings from the literature certainly suggest that subjective experience of chronotype matters. For example, people perform better on cognitive measures when tested at their preferred time of day [e.g., (18)] and perceived exercise exertion varies depending on match between chronotype and the time of day at which exercise is performed (26). Our findings suggest that chronotype defined by objective sleep midpoint may not be equally useful in predicting health behaviors.

Because the difference in executive function disappeared when participants with a sleep disorder were excluded from analyses, it may be that objective and subjective chronotype are not the same construct. Perhaps by measuring sleep midpoint, we captured participants' degree of sleep dysregulation. We argue that sleep timing on its own may have very little to do with health and health behaviors and it may instead be sleep dysregulation that is driving the apparent relationships between chronotype and health.

Should exercise interventionists consider chronotype when trying to maximize engagement? The literature certainly suggests that subjective chronotype may be helpful to take into account in planning activity. However, our findings indicate that objectively captured sleep midpoint is not a useful measure to use independently. Objectively assessed sleep midpoint may be a useful first step in identifying individuals with dysregulated sleep as may have been the case with our evening-type participants. However, it may be more beneficial to consider measures of sleep health (e.g., sleep duration, quality, efficiency) as these clearly predict health outcomes (60) and health behaviors (61).

### Unique contributions and limitations

In addition to the full neuropsychological battery we used to measure cognitive performance, we believe our choice to use actigraphy as our sole measure of chronotype was a strength of our study. This provided an objective measure of participant sleep behavior while simultaneously capturing physical activity in a free-living environment. Actigraphy is widening in popularity due to its many advantages over self-report measures, and so our findings contribute to and expand upon a growing body of literature utilizing body-worn devices to assess health behaviors. That said, actigraphy is a novel approach to measuring chronotype, it has not yet been validated in any population, and our findings suggest that this objective measure of sleep behavior is not a sufficient standalone measure of chronotype. There is a difference between diurnal preference (a subjective construct) and its observable behavioral manifestation (the midpoint of the sleep interval). Older adults more commonly self-report a morning-type preference (10). An individual's sleep midpoint may not actually capture their preferred activity pattern and could instead reflect a concerted effort to adhere to societal demands, which is why subjective measures like the MCTQ use data from non-workdays to calculate chronotype (29). We modeled our sample-specific chronotype classification strategy on a novel approach described by McHugh et al. (21), but we acknowledge that this approach may have limited our ability to effectively test our hypotheses as it yielded a disproportionate number of intermediate-types compared to morning- and evening-types in our sample. Certainly caution is warranted in interpreting many of our findings due to small sample sizes in analyses.

Because our sample consisted of older adults who were primarily retired and able to freely engage in their preferred activity patterns, it is possible that the discrepancy between their actigraphy-calculated sleep midpoint and diurnal preference is smaller than what would be seen in a younger population or those employed full time. Future studies could clarify this distinction by incorporating both an objective and subjective measure of chronotype. A better understanding of the relationship between these two types of assessments and how it varies by age is necessary to inform future research investigating these concepts. For example, the MCTQ sleep midpoint has been shown to correlate with that captured by actigraphy (32), but at this point, it is unclear why our findings suggest that objectively assessed sleep midpoint is not associated with outcomes the way we would expect.

It could also be useful to include a measure of “social jetlag” to determine the degree of mismatch between one's preferred activity patterns and societal demands. Social jetlag is a factor known to associate with health behaviors and health outcomes (e.g., accrued sleep debt), and it is a phenomenon not commonly studied in older adults. If it is demonstrated in this population, it would be important to control for its effects.

Using the ActiGraph GT9X to measure sleep in the absence of any other sleep monitor may also limit the validity of our sleep variables. Studies that investigate actigraphy's ability to measure sleep demonstrate high sensitivity and accuracy, but impaired specificity in that it is unable to differentiate true wake from motionless wake [e.g., (62)]. Our inclusion of sleep diaries may have mitigated some of the concern as participants self-reported time in and out of bed. However, our procedures required manual scoring of sleep when participants did not return their sleep diaries, which was often the case. This manual scoring may have artificially inflated sleep efficiency as we initiated the sleep interval immediately following a dramatic drop in activity levels. We assumed this reflected start time in bed, but it could also indicate sedentary wakeful activity outside of bed (and therefore outside the sleep interval).

This study is limited by its homogenous sample. The participants were primarily Caucasian, highly educated, and motivated to engage in research as evidenced by their participation in a longitudinal study. These characteristics are known to associate with health literacy and engagement in health behaviors (63, 64), thus the findings of our study will not generalize to older adults of differing demographic characteristics. Additionally, many participants enrolled in the KU-ADRC Clinical Cohort opted out of participation in the sub-study from which the proposed study draws its data. Reasons for non-participation included physical limitations, travel plans, no interest in the study, problems with the study design (e.g., lack of feedback to participants), and participants' limited availability. Finally, our sample of participants self-reported the presence or absence of sleep disorders, but this did not yield a well-characterized group or enable complex analyses exploring the effects of sleep disorders on findings.

### Future directions

Ultimately, the present study sought to inform physical activity and sleep intervention research in older adults. We found no differences in total or peak physical activity levels between chronotypes, but we did find that evening-types exhibited significantly worse executive function performance than morning-types, despite morning types more frequently being tested at a less preferred time in our study. This difference in cognitive performance was not apparent when individuals with a sleep disorder were removed from analyses, which suggests that degree of sleep dysregulation rather than sleep timing may actually be driving observed differences. Based on our findings, it appears that an individual's chronotype may have little importance when designing interventions to increase engagement in physical activity in older adults.

## Conclusion

This study used actigraphy and a full neuropsychological battery to study the relationships between physical activity, chronotype, and cognitive performance in an older adult sample of individuals with and without sleep disorders. We found expected differences in daily physical activity patterns between chronotype groups such that morning-types were more active earlier in the day and evening-types showed an opposite pattern. We found no differences in total or peak physical activity levels between chronotype groups, but we did find that evening-types exhibited significantly worse executive function performance than intermediate-types. This latter finding was not apparent when participants with a sleep disorder we excluded from analyses. Future research should clarify the unique influence of objective sleep timing and sleep dysregulation and incorporate both subjective and objective measures of chronotype to determine whether this construct has any meaningful relationship with health behaviors and health outcomes in older adults.

## Data Availability

The dataset presented in this article is not readily available because dataset requests must be made directly to the KU-ADRC. Those interested in accessing the dataset should be directed to the following website where they can complete a data request form: https://www.kumc.edu/research/alzheimers-disease-research-center/research/resources-for-researchers-and-principal-investigators.html.
